# Evaluation of Dual-Band Near-Infrared Spectroscopy and Chemometric Analysis for Rapid Quantification of Multi-Quality Parameters of Soy Sauce Stewed Meat

**DOI:** 10.3390/foods12152882

**Published:** 2023-07-29

**Authors:** Hongzhe Jiang, Yu Zhou, Cong Zhang, Weidong Yuan, Hongping Zhou

**Affiliations:** 1 Jiangsu Co-Innovation Center of Efficient Processing and Utilization of Forest Resources, Nanjing Forestry University, Nanjing 210037, China; 2 College of Mechanical and Electronic Engineering, Nanjing Forestry University, Nanjing 210037, China

**Keywords:** soy sauce stewed meat, near-infrared spectroscopy, fat, protein, chemometrics

## Abstract

The objective of this study was to evaluate the performance of near-infrared spectroscopy (NIRS) systems operated in dual band for the non-destructive measurement of the fat, protein, collagen, ash, and Na contents of soy sauce stewed meat (SSSM). Spectra in the waveband ranges of 650–950 nm and 960–1660 nm were acquired from vacuum-packed ready-to-eat samples that were purchased from 97 different brands. Partial least squares regression (PLSR) was employed to develop models predicting the five critical quality parameters. The results showed the best predictions were for the fat (R_p_ = 0.808; RMSEP = 2.013 g/kg; RPD = 1.666) and protein (R_p_ = 0.863; RMSEP = 3.372 g/kg; RPD = 1.863) contents, while barely sufficient performances were found for the collagen (R_p_ = 0.524; RMSEP = 1.970 g/kg; RPD = 0.936), ash (R_p_ = 0.384; RMSEP = 0.524 g/kg; RPD = 0.953), and Na (R_p_ = 0.242; RMSEP = 2.097 g/kg; RPD = 1.042) contents of the SSSM. The quality of the content predicted by the spectrum of 960–1660 nm was generally better than that for the 650–950 nm range, which was retained in the further prediction of fat and protein. To simplify the models and make them practical, regression models were established using a few wavelengths selected by the random frog (RF) or regression coefficients (RCs) method. Consequently, ten wavelengths (1048 nm, 1051 nm, 1184 nm, 1191 nm, 1222 nm, 1225 nm, 1228 nm, 1450 nm, 1456 nm, 1510 nm) selected by RF and eight wavelengths (1019 nm, 1097 nm, 1160 nm, 1194 nm, 1245 nm, 1413 nm, 1441 nm, 1489 nm) selected by RCs were individually chosen for the fat and protein contents to build multi-spectral PLSR models. New models led to the best predictive ability of R_p_, RMSEP, and RPD of 0.812 and 0.855, 1.930 g/kg and 3.367 g/kg, and 1.737 and 1.866, respectively. These two simplified models both yielded comparable performances to their corresponding full-spectra models, demonstrating the effectiveness of these selected variables. The overall results indicate that NIRS, especially in the spectral range of 960–1660 nm, is a potential tool in the rapid estimation of the fat and protein contents of SSSM, while not providing particularly good prediction statistics for collagen, ash, and Na contents.

## 1. Introduction

Soy sauce stewed meat (SSSM) is a type of representative and traditional Chinese-style ethnic meat product, which is highly praised and well received for its inimitable taste and aroma properties [1]. Generally, SSSM is processed by boiling raw meats with various spices and condiments in water. It is commonly sold in vacuum packaging and is a kind of ready-to-eat convenience product. Owing to its delicious taste and simple processing technique, SSSM is increasingly consumed in China [2].

In the past two decades, its quality, including composition and flavor compounds, has become a research focus in China. In addition to color, texture, flavor, and other external qualities, internal quality traits including protein, fat, ash, collagen, and Na contents are also playing an important role affecting acceptability to consumers and purchase or repurchase decisions [3]. Fat is known to be a major health problem for obesity, and some customers wish to pay for low fat or lean meats only [4]. Protein in meats is beneficial to human health, which provides all types of amino acids needed by the human body and promotes the absorption of calcium. Collagen is the most abundant fibrous protein in mammals, contributing to the tenderness, texture, and sensory characteristics of meats [5]. Ash content is a crucial factor indicating the total inorganic components in meats. Excessive Na intake will lead to hypertension or other cardiovascular diseases including coronary heart disease and stroke [6]. Therefore, there is an urgent necessity to look for a suitable method to comprehensively detect these quality parameters of SSSM.

Sensory analysis is one of the most commonly used methods in meat product quality evaluation and is estimated by trained panelists [7]. However, many subjective factors will influence the final results, including the sampling method, the state of the panelists’ taste perception, and so on. Numerous objective analytical methods are also used, including headspace gas chromatography (HS-GC) [8], gas chromatography-mass spectrometry (GC-MS) [9], solid-phase microextraction (SPME) [10], supercritical fluid extraction (SFE) [11], magnetic resonance imaging (MRI) [12], nuclear magnetic resonance (NMR) [13], etc. These techniques are time-consuming, destructive, or require a series of sample preparation steps. Computer vision (CV) and hyperspectral imaging (HSI) are also frequently used techniques in predicting meat quality [14,15]. However, CV is able to detect only external quality indicators [16], and HSI instruments are too expensive, making them difficult to apply in practice [17,18]. Overall, the above-mentioned methods are not very suitable to meet the high-speed and high-throughput detection needs in the current meat industry.

Near-infrared spectroscopy (NIRS) is a widely applied, convenient, rapid, and non-destructive technique that requires minimal sample processing prior to analysis [19]. The NIR region (780 to 2500 nm) contains information in connection with the relative proportions of C–H, O–H, N–H, and S–H bonds (the primary structural components of organic molecules), mostly due to the overtone vibrational and rotational transitions of these molecular bonds [20]. In addition, NIR light has greater penetration ability than infrared light, making it possible to detect information deep inside samples. Nowadays, a vast number of studies have been conducted by combining chemometrics, including image processing, preprocessing, and modeling, with various optical non-destructive detection techniques [21,22,23,24], and then the quantitative or qualitative performances will be improved. The application of chemometrics to NIR spectra is also helpful to explore the contained relevant information to be used in developing calibration models, and should be evaluated.

With regard to meat quality detection, NIRS has been proven to be able to determine several quality parameters simultaneously, such as protein, fat, moisture, and carbohydrate contents of a wide variety of meats [25]. Also considering the operating speed, NIRS could be a suitable technique for the rapid detection and quality control of meat and meat products. However, to the best of our knowledge, few attempts at the detection of multi-quality parameters in SSSM using NIRS have been made to date.

Therefore, the aim of this study was to introduce a potential methodology of NIRS combining it with chemometric analysis to quantitatively assess several key quality parameters of SSSM. In previous studies, the spectral range of visible and near-infrared region enabled the acquisition of rich spectral information that covers the characteristics of all meat quality attributes [26], and the results have shown that dual-band NIR spectra provided a good foundation for the multi-parameter detection of meats [27]. For this purpose, results for the determination of fat, protein, collagen, ash, and Na contents from dual-band spectral region (650–950 nm and 960–1660 nm) NIRS are presented in the current study. The specific objectives were as follows: (1) evaluate various spectral preprocessing methods applied to full spectra in developing models; (2) compare the predictive ability of spectra in dual-band spectral ranges for predicting different quality parameters; (3) identify quality-parameter-related wavelengths using effective methods; (4) establish multi-spectral simplified models based on the selected variables; and (5) compare the performances of the simplified models with the original full spectra models.

## 2. Material and Methods

### 2.1. Sampling of SSSM

In order to obtain samples with wide variability, a total of 97 pieces of intact SSSM produced by different brands were purchased from JD.com (the B2C e-commerce market in China) (*N* = 52), Taobao Tianmao eco-market (*N* = 32), and local supermarkets (*N* = 13). The samples were all vacuum packed and ready to eat. The brands of the samples included Xi Wang, Qing Cheng, Cao Yuan, Yue Sheng Zai, Yu Run, etc., and the samples were all within their individual shelf life. The production areas of these samples included Inner Mongolia, Shandong, Jiangsu, Xinjiang, Henan provinces, etc. The sizes and masses of the samples varied, including 250 g, 300 g, 350 g, 500 g, and so on. The common ingredients of these products were lean meat, fat, sugar, salt, soy sauce, starch, and spices. The purchased samples in vacuum packaging were delivered or transported to our laboratory, and then stored at 0 to 4 °C in the refrigerator. After that, the samples were individually analyzed within 1 to 10 days of collection.

### 2.2. Physical Analysis and Statistics

Reference quality parameters for the fat, protein, collagen, ash, and Na contents were determined using minced samples in accordance with the official meat analysis methods of standard AOAC [28] procedures. All the collected samples were first individually homogenized using a food blender (S2-A808, Joyoung Co., Ltd., Jinan, China). After that, the fat content was determined by the Soxhlet extraction method using petroleum ether as solvent. The ash content was obtained after burning in a muffle furnace at 550 °C for 16 h or until white or light gray ash was obtained. The protein (i.e., crude protein) content determination procedure can be found in Zheng et al. [29]. The Na content was determined according to a detailed procedure introduced by the reference method in De Marchi et al. [25]. The statistical analysis of the various quality parameters measured, including the Pearson correlation, was all conducted with SPSS software package (SPSS version 19.0, IBM Corporation, Armonk, NY, USA, 2010). The relationships between any two of the five parameters were determined by Pearson correlation analysis with a significance level at *p* < 0.01 or *p* < 0.05.

### 2.3. Spectra Collection

The samples were analyzed by spectroscopy in the visible and near-infrared (VNIR, 400–1000 nm) and near-infrared (NIR, 900–1700 nm) regions in reflectance mode. These dual-band spectra covered the majority of the visible and near-infrared region, which enabled us to acquire rich spectral information that reflects the characteristics of all the meat quality attributes. The minced samples were first stored at room temperature for 30 min (25 ± 3 °C), and then spectra were collected in three different positions (randomly selected) of the ground meats with a flat surface. Two spectrometers were individually employed in this study to collect spectra in the same positions. The VNIR spectra were collected using an Ocean Optics USB2000+ spectrometer (Ocean Optics Ltd., Dunedin, FL, USA) working in spectral range of 399.8 to 949.8 nm in the laboratory. The NIR spectra were obtained using a Hamamatsu S9214-512 spectrometer (Beijing Hamamatsu Photonics Technology Ltd., Beijing, China) working in spectral range of 942.7 to 1698.3 nm. The spectrometers were interfaced to a personal computer (Lenovo Tianyi 510 Pro, Lenovo Group Ltd., Beijing, China) and controlled by their individual operational software. The spectra collection was conducted by two spectral acquisition setups through two optical fiber probes at each position simultaneously. The sampling intervals were 0.35 nm and 3.6 nm, respectively, and the spectra were averaged from three repeat measurements. Finally, high-noise wavebands of three spectral regions (399.8–650 nm, 942.7–960 nm, and 1650–1698.3 nm) were excluded.

### 2.4. Spectral Preprocessings

Prior to the model development, various chemometric spectral preprocessings were evaluated, aiming to reduce undesired information including standard normal variate (SNV), SNV and detrend (SNVD), and derivatives. SNV was applied to remove the path length variations due to a light scattering effect [30]. SNVD was employed to suppress the baseline shifting and curvilinearity [31]. Derivatives were applied to separate overlapping peaks or valleys of the spectra and to reduce the influence of random noises. The first-order and second-order derivatives (Der1 and Der2) were implemented with the Savitzky–Golay algorithm with a second-order polynomial within a moving window of 15 points. All the chemometric analysis in this study was conducted in the Unscrambler software (version X10.1, CAMO ASA, Oslo, Norway).

### 2.5. Model Development

Partial least squares regression (PLSR) is emerging as one of the most robust and reliable chemometric modeling methods when variables are highly collinear. It is known that NIR spectra have a serious collinearity problem with so many points in the spectral dimension, and PLSR is an effective method to describe the latent relationship among spectral variables, which is commonly applied in spectral analysis. PLSR allows spectral information to be converted into latent variables (LVs), which can be used to describe the maximum covariance between the spectral data and reference quality parameters [32]. PLSR transforms both spectra (matrix *X*) and responses (matrix *Y*) to an LV space first. One of the most significant points lies in the fact that matrix *X* is decomposed into a linear combination of scores (*T*) and loadings (*P*) matrices considering the given response matrix *Y*. Then, in the calibration step, leave-one-out cross-validation (LOOCV) was employed to identify the optimum number of LVs [33]. The establishment of the PLSR models was carried out using MATLAB software (R2013b, The MathWorks Inc., Natick, MA, USA).

### 2.6. Effective Wavelength Selection

As is known, many continuous wavelengths present redundant information in modeling and should be reduced. To overcome this drawback, effective wavelength selection is usually performed. Regression coefficients (RCs) and random frog (RF) algorithms were employed, and the effectiveness comparison was evaluated in this study.

RCs (i.e., *β*-coefficients) resulting from the preferred PLSR calibration model were used for choosing the effective wavelengths. In RCs, wavelengths corresponding to the highest absolute values of the coefficients are considered to make a greater contribution to PLSR modeling, and are obtained. Moreover, the lowest absolute values of the coefficients are completely neglected because they make little or no contribution to the predictions.

RF was also carried out based on the reversible jump Markov chain Monte Carlo framework. The main RF selection steps involved are as follows. First, a subset of variables was generated from the initial variables. After that, a new candidate subset was randomly generated to update the initialization subset. Finally, the selection probability was calculated for each variable after the predefined N iterations. Generally, the more important a variable is, the more likely it is to be chosen and retained in a new subset. In our study, the number for *N* was set to 1000 and wavelengths with a high selection probability were applied as important ones. The RC and RF procedures were individually operated using written programs in the Matlab software.

### 2.7. Model Performance Evaluation

The performance of the developed models was evaluated using the parameters of correlation coefficient (R), root mean squared error (RMSE), and residual predictive value (RPD). The correlation coefficient of calibration (R_c_), cross-validation (R_cv_), and prediction (R_p_), as well as the root mean squared error in calibration set (RMSEC), cross-validation set (RMSECV), and prediction set (RMSEP), were individually calculated. The RPD in prediction set was evaluated by calculating the ratio of standard deviation (SD) to RMSEP. RPD has been employed as an indicator of the reliability of calibration models in many studies [34]. If the RPD value was greater than 2.5, the model would be considered good for calibration. And RPD above 5 was considered adequate for analytical purposes, whereas values lower than 1.5 demonstrated that the prediction was inadequate [35]. The R and RPD are dimensionless and unitless numbers. In particular, models with high R and RPD and low RMSE indicated a good practical utility.

## 3. Results and Discussion

### 3.1. Spectral Profiles

**Figure 1:**
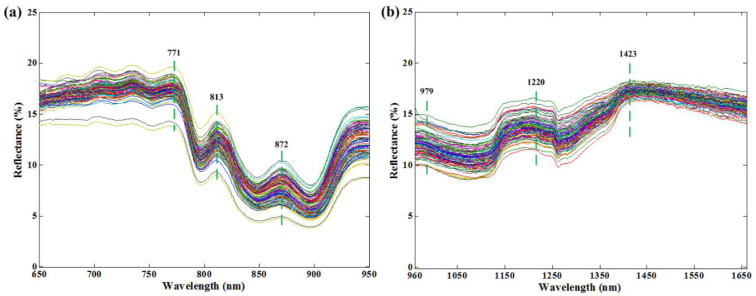
Mean spectral profiles of SSSM samples in spectral range of (**a**) 650–950 nm, and (**b**) 960–1660 nm.

The dual-band original spectra of all the measured samples are shown in Figure 1. As can be seen, the average spectra showed very high similarity among the different samples regardless of the spectral regions. This is because the SSSM had similar chemical composition. Several spectral broad bands can be clearly found, including 771 nm, 813 nm, 872 nm, 979 nm, 1220 nm, and 1423 nm. The 771 nm band was closely related to the third overtone of the O–H stretching mode of water [36]. The 979 nm band was due to the water absorption band related to the O–H stretching second overtones [37,38]. It was indicated that moisture dominated these two spectral characteristics [39]. The 1220 nm band was associated with the second overtone of the symmetric stretching mode of C–H related to the fat molecules [40,41]. The band at 813 nm was also related to the fat content [42]. A significant peak of 1423 nm was observed that indicated the combination of the C–H stretching and vibration with other vibration modes of the concerned molecule [43]. Another reflectance peak at 872 nm was connected with the C–H stretching vibration of aliphatic compounds [44]. Overall, these characteristic wavelengths might contribute some significant information to predicting multi-quality parameters in SSSM samples.

### 3.2. Statistics of Reference Analysis

The descriptive statistics of measurement results of the fat, protein, ash, collagen, and Na contents determined by the reference methods in all the 97 samples from different brands are displayed in Table 1. A randomly divided calibration set (70 samples) and prediction set (27 samples) with their similar means, ranges, coefficient of variation (CV), and standard deviations (SDs) for all the quality parameters studied are also displayed. As shown, there was a relatively high variability observed in these quality parameters (CV was greater than 18% regardless of calibration set or prediction set) which was attributed to the extensive sample sources of all the well-known SSSM brands on sale in Chinese markets. The reasonable variability of the quality parameters of the samples was quite beneficial for establishing a universal and stable regression model. For example, the values covered a large span from 16.5 g/kg to 162.5 g/kg, indicating that the variation was enough to allow a correct calibration. The small differences in means, ranges, and SDs between the calibration set and the prediction set indicated these two sets were representative of the similar overall diversity [45]. Once the calibration and prediction sets had been established, the prediction models were further developed for all five of the quality indicators.

The Pearson correlation coefficients between any two quality parameters are shown in Table 2. It can be seen that the fat content was unrelated to the other four parameters, which showed very weak Pearson correlation coefficients ranging from −0.124 to −0.021. The values for the protein content were positively correlated to the collagen content (*p* < 0.01), and negatively correlated to the ash content (*p* < 0.05) and Na content (*p* < 0.01). No significant correlations were found between the collagen content and the ash or Na contents. The correlation coefficient between the ash content and Na content was higher than 0.76 (*p* < 0.01). This suggested that ash content could be used to roughly indicate Na content, since both of them are inorganic substances.

### 3.3. Correlation between Spectra and Quality Parameters

**Figure 2:**
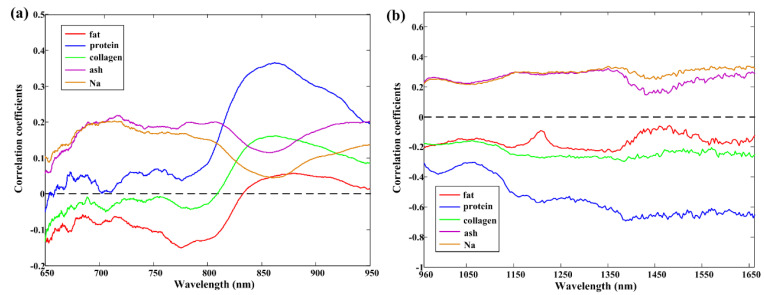
Correlation coefficients between each quality parameter (fat, protein, collagen, ash, or Na) and (**a**) VNIR or (**b**) NIR spectra.

The curves of the correlation coefficients between the dual-band raw spectra and five individual quality parameters (fat, protein, collagen, ash, or Na contents) at each wavelength are individually shown in Figure 2. It is well known that the higher the absolute value of the correlation coefficient, the greater the strength of the variable in predicting quality parameters [46]. The curves showed similar general patterns for the first three quality parameters (fat, protein, and collagen) and for the last two quality parameters (ash and Na), regardless of whether they were in the VNIR or NIR spectral region. The absolute correlation coefficients ranged from 0 to 0.2 for the fat content in both waveband ranges. As for the protein content, 960–1660 nm performed better than 650–950 nm, with absolute values ranging from 0 to 0.3 and 0.2 to 0.8, respectively. The highest absolute values ranged from 0.6 to 0.8 in the waveband located from 1400 nm to 1660 nm. The main reason was that the wavelengths around 1400 nm were connected with the first overtone of O–H and N–H in the amino and amide groups relating to the protein content [47]. Regarding the correlation coefficients for the collagen content, the absolute values were less than 0.3 whether in the VNIR or NIR regions. The ash curve and Na curve had similar correlation coefficients, ranging from 0 to 0.2 in the VNIR region and from 0 to 0.3 in NIR region.

### 3.4. Evaluation of PLSR Predictive Models

PLSR analysis was conducted using the dual-band full spectra (650–950 nm and 960–1660 nm, respectively) to develop models for the predictions. The statistical results of the R, RMSE, and RPD values in the calibration, cross-validation, and prediction sets of the optimal models for individually predicting the fat, protein, collagen, ash, and Na contents of the SSSM are summarized in Table 3. The large number of preprocessing attempts enabled us to explore the best performance in the calibration, cross-validation, and prediction sets, and the optimal preprocessing methods are also displayed in the table.

As shown in Table 3, the quality parameters were generally predicted better by the spectra in the waveband range of 960–1660 nm than by the VNIR spectral data in the range of 650 to 950 nm. For the 650–950 nm range, it can be seen that the R_p_ (0.242 to 0.617), RMSEP, and PRD (0.936 to 1.193) values revealed a less strong relationship between the spectra and various quality parameters. As for the waveband of 960–1660 nm, better performances in calibration, cross-validation, and prediction were observed for the fat and protein (R_p_ = 0.808 and 0.863) contents than for the collagen, ash and Na contents (R_p_ = 0.403, 0.206, and 0.201). However, it is noted that the collagen, ash and Na contents exhibited higher prediction errors than the other two quality parameters regardless of whether the waveband was 650–950 nm or 960–1660 nm. For example, the optimal model for the Na content prediction was obtained by using SNVD in the spectral range of 650–950 nm, and the prediction results yielded R_p_ = 0.242 and RMSEP = 2.097 g/kg. In addition, the RPD value was low (RPD = 1.042), indicating that the model was unacceptable for Na content screening. The lack of acceptable prediction ability for collagen, ash, and Na contents of meat or meat products is not new. Several studies for ash and collagen predictions in meats showed similar results; for example, Alomar et al. [48] reported that the prediction ability of calibrations was poor for total ash content and very poor for collagen content in beef.

The reason for the unsuccessful prediction of the ash and Na contents of the SSSM was that NIR spectroscopy does not interact with inorganic compounds or pure minerals in their ionic forms [49]. In addition, Na content predictions in prior publications [50,51] were better than the results obtained in our work. This might be because although the inorganic substance of salt (determined Na content) did not absorb in the near-infrared region, it was related to the organic fraction through the associations with organic acids, or chelates. However, the soy sauce coatings on the surface of the samples might influence the absorption. Alternative spectroscopic techniques or new modeling methods could be further developed to try to make successful predictions for collagen, ash, and Na contents.

The fat and protein prediction results were comparable to other previous studies connected with meats or meat products. Alomar et al. [48] reported that the optimal results achieved for the fat and protein contents of bovine meat were coefficients of determination (R_cv_^2^) and standard error (SECV) in cross-validation sets equal to 0.84 and 0.82, and 0.44 and 0.48, respectively. Wang et al. [26] found high correlations between NIR spectra and fat as well as protein contents (R_p_ = 0.93 and 0.86, RMSEP = 0.647 g/kg and 1.033 g/kg) for pork. However, several other studies reported unreliable prediction results of R_p_^2^ (coefficient of determination in prediction) ranging from 0.2 to 0.5 for fat content [52,53], and protein content [54,55]. The main reason might be the unsuitable spectral range or the low variability of the original fat or protein contents of the meat samples. Finally, the 960–1660 nm range was selected for further analysis in determining only fat and protein contents. Concerning the spectral preprocessings, the optimal ones for fat and protein were both Der1, which was adopted in further analysis.

### 3.5. Selection of Sensitive Wavelengths

**Figure 3:**
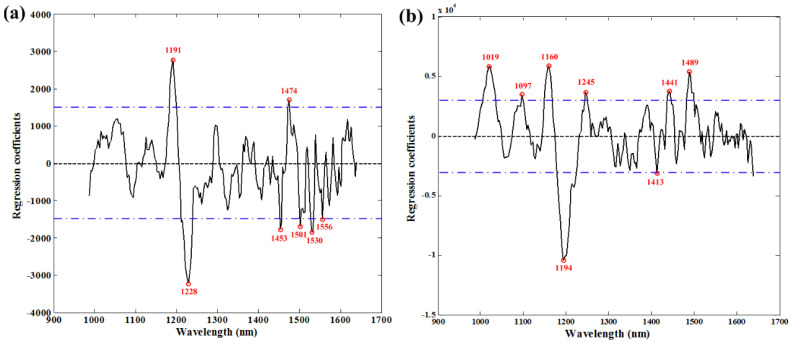
Selection of wavelengths using RCs of the optimal PLSR models for (**a**) fat and (**b**) protein.

The NIR spectrum provides a kind of high-dimensional spectral data, and has its own limitations in practical application. The use of fewer spectral variables is preferable for more stable models and easier implementation in subsequent multi-spectral systems. In order to reduce the large dimensionality and avoid redundancy among contiguous bands, quality-related wavelengths were selected from the developed optimal PLSR models [56]. The weighted RCs that resulted from the optimal PLSR models were considered as the various quality-parameter-related wavelengths. By means of this approach, the peaks and valleys corresponding to high absolute values for fat and protein were identified in Figure 3, respectively. Overall, seven and eight wavelengths were finally obtained for further simplified model development. Furthermore, RF was also carried out to select key wavelengths, and ten variables were retained. All the wavelengths selected using RCs and RF are shown in Table 4. These wavelengths will be applied to simplify the PLSR models.

### 3.6. Multi-Spectral Model Development

**Figure 4:**
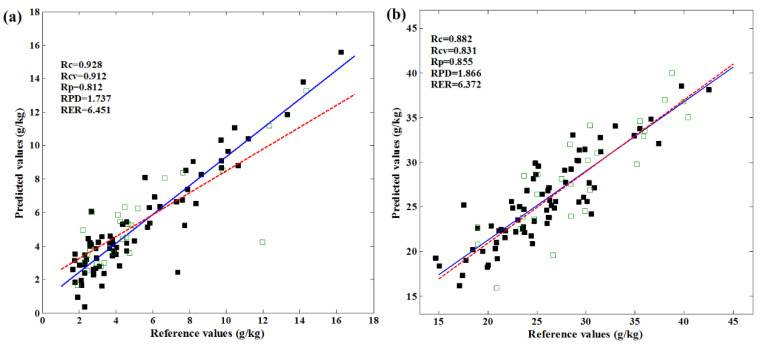
Graphical relationships between reference values and predicted values of the preferred prediction models for (**a**) fat and (**b**) protein. (■ Calibration set; □ Prediction set; “**—**” regression line of calibration; “**---**” regression line of prediction).

The performances of the multi-spectral PLSR models based on selected wavelengths by RCs and RF are shown in Table 5. The RC-PLSR model for predicting fat content showed R_p_ = 0.775, RPD = 1.475, and RMSEP = 2.274 g/kg. Similarly, the RF-PLSR model using ten selected wavelengths attained slightly better accuracy with R_p_, RMSEP, and RPD of 0.812, 1.930 g/kg, and 1.737, respectively. The RC-PLSR model for predicting protein content achieved good accuracy with R_p_, RMSEP, and RPD of 0.855, 3.367 g/kg, and 1.866, respectively. The RF-PLSR model for protein prediction also performed well; however, the result showing an RPD value of 1.694 had a lower performance than the RC-PLSR model. This model was also able to be applicable for rough screening. To visualize the robustness of the optimal models graphically, the plots of the predicted fat or protein contents against their reference values are displayed in Figure 4. As shown, all the samples can be predicted to be near their individual reference value.

Generally, these simplified models showed a comparable performance with the original models based on the full spectra shown in Table 3. It indicated that the selected variables carried sufficient information in developing simplified models. The results indicated that wavelengths at the NIR measures are closely related to the characteristics of SSSM quality including fat and protein. Based on the evaluation, the selected two models (RF-PLSR for fat content and RC-PLSR for protein content) can be implemented for further application.

In the literature, predictions have been widely investigated for these two quality parameters in a variety of meat or meat products such as beef, pork, and poultry using VNIR or NIR spectroscopy. However, the performances were not always consistent. As indicated in Section 3.4, this may be due to many factors, including sampling numbers, spectral ranges, sampling variability, preprocessings, etc. The results concluded that the spectra can be helpful in calculating approximate content, but are not fully reliable for achieving a good performance in predicting exact content. The soy sauce in the SSSM had a certain impact on the prediction results, leading to an acceptable rather than excellent performance.

## 4. Conclusions

In the present paper, the performance of VNIR (650–950 nm) and NIR (960–1660 nm) spectroscopy techniques combined with chemometrics for determining five key quality parameters of SSSM were evaluated. The calibration models in the spectral range of 960–1660 nm produced PLSR models with good R_p_ (0.808 and 0.863) and RPD (1.666 and 1.863) for the fat and protein contents, respectively. The collagen, ash, and Na contents were not predicted so well, mainly because the NIR spectra did not interact with the inorganic compounds or pure minerals and the soy sauce coatings also influenced the performance. The RCs and RF were successfully utilized for wavelength selection, and, through comparison, RF-PLSR for fat and RC-PLSR for protein were regarded to be the optimal multi-spectral models. These results suggest that NIR spectroscopy has considerable potential to predict the fat and protein contents of SSSM. Given the success of this current work, the expansion of this study is also highly recommended. Further research on these effective calibration models will include transferring these laboratory-based models to an industrial environment. Probably, further NIRS models could also be employed to estimate quality traits, shelf life, etc., of other meats or food matrices.

## Figures and Tables

**Table 1:** Descriptive statistics of quality parameters for calibration set and prediction set.

Parameters	Calibration Set (70 Samples)				Prediction Set (27 Samples)			
	Mean	SD	Range	CV (%)	Mean	SD	Range	CV (%)
Fat (g/kg)	51.2	33.2	16.5 to 162.5	64.8	50.5	33.5	19.0 to 143.5	66.4
Protein (g/kg)	256.2	56.4	146.5 to 425.5	22.0	294.5	62.8	189.5 to 404.0	21.3
Collagen (g/kg)	26.1	18.6	0.1 to 104.0	71.1	32.2	18.5	1.8 to 90.5	57.3
Ash (g/kg)	28.0	5.3	12.0 to 40.5	18.9	27.4	5.0	18.0 to 35.0	18.2
Na (g/kg)	8.5	2.4	1.3 to 16.2	28.8	8.1	2.2	2.6 to 12.1	27.1

**Table 2:** Pearson correlation coefficients of quality parameters of soy sauce stewed meat samples.

Parameters	Fat (g/kg)	Protein (g/kg)	Collagen (g/kg)	Ash (g/kg)	Na (g/kg)
Fat (g/kg)	1				
Protein (g/kg)	−0.078	1			
Collagen (g/kg)	−0.021	0.353 **	1		
Ash (g/kg)	−0.142	−0.218 *	0.038	1	
Na (g/kg)	−0.124	−0.282 **	0.043	0.769 **	1

** *p* < 0.01, * *p* < 0.05.

**Table 3:** Performance of PLSR prediction models using full spectra with optimal preprocessings.

Wavebands	Traits	Preprocessings	LVs	Calibration Set		Cross-Validation Set		Prediction Set		RPD
				R_c_	RMSEC (g/kg)	R_cv_	RMSECV (g/kg)	R_p_	RMSEP (g/kg)	
650–950 nm	Fat	Der1	3	0.487	2.880	0.070	3.543	0.382	3.573	0.938
	Protein	Der1	4	0.742	3.755	0.503	4.945	0.617	5.264	1.193
	Collagen	Der1	8	0.782	1.150	0.157	2.131	0.524	1.970	0.936
	Ash	Der1	2	0.470	0.466	0.075	0.564	0.384	0.524	0.953
	Na	SNVD	3	0.310	2.308	0.032	2.541	0.242	2.097	1.042
960–1660 nm	Fat	Der1	7	0.967	0.842	0.912	1.366	0.808	2.013	1.666
	Protein	Der1	9	0.951	1.731	0.824	3.214	0.863	3.372	1.863
	Collagen	SNV	3	0.481	1.617	0.257	1.818	0.403	1.666	1.108
	Ash	none	3	0.346	0.495	0.107	0.540	0.206	0.501	0.997
	Na	none	2	0.290	2.323	0.109	2.453	0.201	2.123	0.976

**Table 4:** Wavelengths selection by various methods.

Methods	Traits	Numbers	Selected Wavelengths (nm)
RCs	Fat	7	1191, 1228, 1453, 1474, 1501, 1530, 1556
	Protein	8	1019, 1097, 1160, 1194, 1245, 1413, 1441, 1489
RF	Fat	10	1048, 1051, 1184, 1191, 1222,1225, 1228, 1450, 1456, 1510
	Protein	10	1019, 1150, 1153, 1191, 1194, 1198, 1255, 1259, 1285, 1629

**Table 5:** Performance of multi-spectral PLSR models using selected wavelengths.

Traits	Models	LVs	Calibration Set		Cross-Validation Set		Prediction Set		RPD
			R_c_	RMSEC (g/kg)	R_cv_	RMSECV (g/kg)	R_p_	RMSEP (g/kg)	
Fat	RC-PLSR	5	0.919	1.299	0.884	1.550	0.775	2.274	1.475
Protein	RC-PLSR	6	0.882	2.639	0.831	3.122	0.855	3.367	1.866
Fat	RF-PLSR	3	0.928	1.229	0.912	1.357	0.812	1.930	1.737
Protein	RF-PLSR	5	0.885	2.602	0.828	3.146	0.870	3.708	1.694

## Data Availability

The data presented in this study are available on request from the authors.
